# Hib Vaccines: Their Impact on *Haemophilus influenzae* Type b Disease

**DOI:** 10.1093/infdis/jiaa537

**Published:** 2021-09-30

**Authors:** Janet R Gilsdorf

**Affiliations:** Department of Pediatrics, University of Michigan Medical School, Ann Arbor, Michigan, USA

**Keywords:** *Haemophilus influenzae*, vaccine, meningitis, PRP

## Abstract

*Haemophilusinfluenzae* serotype b (Hib) is an important cause of serious, invasive infections, particularly in young children. Since 1985, a series of vaccines composed of the type b capsular polysaccharide polyribosylribitol phosphate (PRP), followed by PRP conjugated to various proteins, have been licensed for use in the United States and worldwide. The conjugated vaccines offer increased immunogenicity and prolonged durability of immune protection compared to the plain PRP vaccine and increasingly are combined with other childhood vaccines for decreased cost and increased ease of vaccination. Hib vaccines have a very favorable safety profile, have been found to be either cost-saving or cost-effective by many public health agencies, and, in most countries, are initiated during early infancy as part of routine childhood immunization programs. As a result of widespread use of the vaccines, the incidence of Hib infections, and their associated morbidity and mortality, has fallen dramatically across the globe. Yet, many children remain unimmunized or underimmunized against Hib, particularly in limited-resource countries. Future efforts to further reduce the disease burden of Hib infections remain a high priority.

The encapsulated, gram-negative bacterium *Haemophilus influenzae*, serotype b (Hib), causes serious invasive infections, such as meningitis, septic arthritis, bacteremia, facial cellulitis, and epiglottitis in nonimmune individuals, and are particularly prevalent in young children. Early studies by Fothergill and Wright documented that children aged 3 months to 5 years are uniquely susceptible to Hib meningitis because they lack bactericidal activity (now known to be mediated by type b capsule-specific antibodies) in their blood [1]. Newborns, on the other hand, are protected by maternal antibodies, and older children and adults possess naturally acquired, protective antibodies.

Prior to the introduction of the Hib vaccine, approximately 1 in 200 young children in the United States developed Hib invasive infection before their fifth birthday annually, representing 12 000 cases of Hib meningitis per year [2]. The annual incidence of Hib meningitis among children <5 years of age ranged from 19 to 69 per 100 000 [3]; this incidence was considerably lower in Europe, South America, Asia, and Oceana but much higher among aboriginal children in the United States (American Indians and Alaska Natives), Canada, New Zealand, and Australia [4]. These differences may be explained by variations in reporting, by environmental conditions such as household crowding, and by biologic differences among children of different ethnicities.

Bacterial meningitis is often a devastating infection, and outcomes of children with this disease vary, dependent on the causative organism and on the availability of medical care [5]. Death from meningitis in developed countries as reported in 1993 was 4.5% and in undeveloped countries was 9.1%. Neurological sequelae of *H. influenzae* meningitis, on the other hand, were similar in all countries studied and included mental retardation (6.1%), spasticity/paresis (5.1%), seizure disorder (6.1%), hearing loss (10.2%), and severe hearing loss (6.7%); only 73.9% of the patients had no sequelae [5]. Moreover, long-term follow-up studies showed behavioral and/or educational disorders in 45% of children with Hib meningitis [6]. Shortly before availability of effective Hib vaccines, mortality in patients with *H. influenzae* meningitis in the United States ranged from 3% to 6% [7, 8].

## HIB VACCINES

The first vaccine against Hib, which consisted of the type b purified polysaccharide capsule polyribosylribitol phosphate, or PRP [9], was modeled after the capsular vaccines against *Streptococcus pneumoniae* [10] and *Neisseria meningitidis* [11]. Long-term protection against Hib disease was shown to be associated with PRP antibody levels of ≥0.15 μg/mL in unvaccinated individuals and ≥1.0 μg/mL in children recently vaccinated with purified PRP [12, 13]; such protective levels do not necessarily apply to children immunized with Hib conjugate vaccines, which generate more robust and enduring immune responses. Unfortunately, children under age 18 months, a major risk group, demonstrated poor immunologic responses to the plain PRP vaccine and were not well protected from Hib disease. Although the efficacy of the PRP vaccine was variable in the United States [14], it was licensed for children over age 18 months on the basis of a large study in Finnish children [15]. Considerable controversy surrounded the appropriate use of this vaccine based on regional differences in efficacy and on various interpretations of the data [14]. Subsequently, PRP alone was soon replaced by a series of Hib vaccines in which PRP was conjugated to various proteins to improve the immunogenicity in young children.

Building on the work of Landsteiner and van der Scheer, who showed that immune responses to optical isomeric acids were enhanced when the acids were combined with proteins [16], and that of Avery and Goebel, who showed that pneumococcal serotype III capsular antigen was more immunogenic in rabbits when conjugated to horse globulin [17], 2 groups of investigators (John Robbins/Rachel Schneerson at the National Institute of Child Health and Human Development and David Smith/Porter Anderson at Harvard Medical School), as well as scientists at Merck Sharp & Dohme Research Laboratories, conjugated purified Hib capsular polysaccharide to various proteins and demonstrated increased immunogenicity in infants [18–20]. The increased immunogenicity of the conjugated polysaccharides rests with the T-cell dependency of their immune responses, while responses to pure polysaccharides are T-cell independent, characterized by their poor immunogenicity in children under age 2 years and poor immunologic memory [21]. Thus, conjugated polysaccharide vaccines promote increased Hib antibody levels in children, the induction of memory cells, and a booster response upon the vaccinee’s subsequent exposure to the type b antigen.

To date, 4 Hib conjugate vaccines have been developed, each with polysaccharides of different lengths and configurations as well as with polysaccharides conjugated to different carrier proteins using different conjugation processes; thus, each demonstrates unique immunologic properties [22] (Table 1).

**Table 1.** *Haemophilus influenzae* Type b Vaccines

Vaccine	Brand Name	Manufacturer	Carrier Protein
Monovalent			
PRP	B-CAPSA 1	Praxis	None
	HibImmune	Lederle	None
	HibVAX	Connaught	None
PRP-D	ProHIBiT	Connaught	Diphtheria toxoid
HbOC	HibTITER	Praxis	CRM197
PRP-OMP	PedvaxHIBa	Merck & Co, Inc	*Neisseria meningitidis* outer membrane protein
PRP-T	ActHIB^a^	Sanofi Pasteur	Tetanus toxoid
	OmniHIB	Sanofi Pasteur	Tetanus toxoid
	Hiberix^a^	GlaxoSmithKline	Tetanus toxoid
Combination			
PRP-T, DTaP	Actacel	Sanofi	
HbOC, DTP	Tetrammune	Lederle	
PRP-OMP, HepB	Comvax	Merck & Co, Inc	
PRP-T, DTaP, IPV	Pentacel^a^	Sanofi Pasteur	
PRP-T, MenAY	MenHibrix	GlaxoSmithKline	
PRP-OMP, DTaP, IPV, HepB	Vaxelis^b^	Sanofi Pasteur & Merck & Co, Inc	

Abbreviations: DTaP, diphtheria, tetanus, acellular pertussis; DTP, diphtheria, tetanus, pertussis; HbOC, Haemophilus b oligosaccharide conjugate; HepB, hepatitis B; IPV, inactivated polio vaccine; MenAY, meningococcal types A and Y; PRP, polyribosylribitol phosphate; PRP-D, polyribosylribitol phosphate-diphtherai; PRP-OMP, polyribosylribitol phosphate-outer membrane protein; PRP-T, polyribosylribitol phosphate-tetanus toxoid.

^a^Currently available in the United States.

^b^Available 2020–2021 in the United States.

### PRP-D

The first Hib conjugate vaccine, licensed in the United States in 1987, contained PRP conjugated to diphtheria toxoid (PRP-D). This vaccine showed immunogenicity and protection against invasive Hib disease in Finnish children older than 18 months [23, 24] and was subsequently recommended for use in the United States in children at 18 months of age. Because of its relatively low immunogenicity in very young children [25, 26], it has been replaced by newer PRP conjugate vaccines.

### HbOC (Also Known as PRP-CRM)

Based on studies of excellent immunogenicity [27], HbOC—PRP conjugated to CRM_197_, a nontoxic, mutant variant of diphtheria toxin—was licensed in 1988 by Praxis and recommended in 1990 for routine use in children at 2, 4, and 6 months of age with a booster at 15–18 months. After several buyouts by successive manufacturers, in 2007, changes in business priorities led the final manufacturer to withdraw this product from the market.

### PRP-OMP

PRP-OMP—PRP conjugated to outer membrane proteins contained in bacterial vesicles from Neisseria meningitidis—was licensed in 1989 in the United States. Because it does not require a priming dose but, rather, stimulates a strong immune response after the first dose [28], it is recommended to be given at 2 and 4 months of age, with a booster dose at 15–18 months and is the preferred vaccine for children at risk of Hib infection at a very young age, such as indigenous children in the United States, New Zealand, and Australia.

### PRP-T

In 1993 PRP conjugated to tetanus toxoid (PRP-T) ActHIB, was licensed in the United States [29]. In 2009 a second PRP-T product, Hiberix was licensed by the United States Food and Drug Administration (FDA) for use only as the booster dose. Based on noninferiority to ActHIB, in 2016 the recommendation was expanded to also use Hiberix in the primary, 3-dose series [30]. This vaccine, in combination with other childhood vaccines, is widely used in resource-poor countries.

### Combination Vaccines

A number of products containing PRP conjugate vaccines combined with other childhood vaccines have been licensed for use in the United States, and only Pentacel (PRP-T, diphtheria, tetanus, acellular pertussis [DTaP], inactivated polio vaccine [IPV], and hepatitis B [HepB]) is currently available; Vaxelis (PRP-OMP, DTaP, IPV, HepB) is anticipated to be available in the United States in 2020 or 2021. Additional pentavalent and hexavalent combination PRP conjugate vaccines are prequalified by the World Health Organization (WHO) for global use, such as the high-priority diphtheria, tetanus, whole cell pertussis (DTwP)–HepB-Hib-IPV. The advantages of combination over monovalent vaccines include fewer injections, fewer office visits for administration of routine childhood vaccines, and potentially lower cost.

## HIB VACCINE IMMUNOGENICITY AND EFFICACY

The efficacy of conjugated Hib vaccines relies on functionality of the antibodies they induce, and thus depends on the levels of serum antibody in vaccinated individuals, their isotypes (immunoglobulin G1–G4, immunoglobulin M, and immunoglobulin A), the avidity of antibody binding to the vaccine antigen, and their form related to presentation of the target antigen [31].

Comparative immunogenicity studies of Hib conjugate vaccines that calculated the percentage of infants with ≥1 μg/mL capsular antibody, which is thought to be important for long-term protection, following the infant series showed PRP-T = 83% of vaccinees, HbOC = 75%, PRP-OMP = 55%, and PRP-D = 29% [25]. In vaccinated, very low birth weight infants, 79% demonstrated anti-PRP levels ≥1 μg/mL [32]. Other immunogenicity studies showed significantly increased antibody to PRP-OMP after only 1 and 2 doses [33, 34]. This unique immunogenicity pattern renders PRP-OMP particularly useful in protecting Native Alaskan children [35] and American Indian children [36], who, for unknown reasons, develop Hib infection at younger ages than other children in the United States [37–39]. Combination vaccines, composed of PRP conjugates used in licensed monovalent vaccines plus other childhood vaccines, elicit, in general, similar anti-Hib antibody responses as the monovalent products [40], and their use does not interfere with adequate responses to the other childhood vaccines [41].

Efficacy studies of the first Hib conjugate vaccine, PRP-D, in Finland showed 89% protection following 2 doses and 100% protection following the booster dose [23], and although no efficacy trials were reported prior to FDA licensure of PRP-D in the United States, 4 postlicensure studies demonstrated high efficacy among children 15–60 months of age [42]. In Northern California, efficacy of HbOC after the first and second doses at 2 and 4 months and at 18 months of age was 26%, 100%, and 100%, respectively [27]. In Southern California, efficacy of HbOC after the first, second, and third doses starting at age 2 months was 71.1%, 88.8%, and 94.4%, respectively [43]. Efficacy of PRP-T at ages 2, 3, and 4 months in the United Kingdom showed efficacy of 95% [44]. PRP-OMP efficacy studies were not conducted prior to FDA licensure, but postlicensure studies have demonstrated high efficacy [36, 45]. Thus, HbOC, PRP-T, and PRP-OMP offered excellent immunogenicity and efficacy in children as young as 2–6 months of age.

Among Alaskan children, efficacy of PRP-D after the first, second, and third doses was 25%, 35%, and 43%, respectively [46]. On the other hand, because of its unique immunogenicity dynamics, the efficacy at 18 months of age of PRP-OMP given at age 2 and 4 months among Navajo and Apache children in Arizona was 95% [36].

Hib invasive infections occasionally occur despite adequate immunization for Hib. Among children exhibiting such apparent vaccine failures at <1 year of age, 44% had either a clinical risk factor (such as prematurity, malignancy, developmental delay, Down syndrome, or neutropenia) or an immunodeficiency or both; among those with vaccine failure vaccinated at >1 year of age, 67% had either a clinical risk factor or an immunodeficiency or both [47]. Children without a clinical risk factor who experienced Hib vaccine failure demonstrated higher levels of anti-PRP antibody following the Hib infection than healthy control children, but the antibody avidity was significantly lower [48]. The mean anti-tetanus antibody level and avidity index for children with vaccine failure and healthy control children were similar. These results suggest that vaccine failures result from inadequate immunological priming of polysaccharide antigens, leading to impaired avidity maturation of Hib-specific B cells or to lack of, or loss of, B cells that produce high-avidity antibody, despite evidence that priming had permitted significant antibody responses following invasive Hib disease.

## HIB VACCINE SAFETY

Early studies of monovalent Hib conjugated vaccines demonstrated mild to moderate local reactions (pain, swelling, erythema) in 13%–30% of recipients [25]. Recent data from the Vaccine Adverse Events Reporting System, which contains reports from 1990 to the present, documented 47 354 events for all Hib-containing vaccines and 829 deaths. Among the most frequently reported adverse events were fever (32.6%), crying and screaming (19.3%), injection site reaction (19.2%), rash (18.4%), and agitation and irritability (15.7%) [49]. Combination vaccines showed higher rates of fever and local reactions, as well as irritability on the day of injection, but these differences were not thought to be clinically significant [40, 41].

Although 33 cases of Guillain-Barré syndrome and 100 cases of thrombocytopenia were associated with receipt of any PRP vaccine, an early report from the Institute of Medicine could not ascribe Hib vaccine causality to such cases [49]. Furthermore, evidence was inadequate to accept or reject a causal relation between Hib vaccines and sudden infant death syndrome or non–Hib infection death [50].

## HIB VACCINE USE

Conjugated Hib vaccines are used widely in developed countries and are increasingly used in limited-resource countries. Different vaccine schedules are used globally, such as 2 primary doses + 1 booster, 3 primary doses with no booster, and 3 primary doses + 1 booster, and the initial primary dose may begin at 6 or 8 weeks of age. Results of meta-analysis studies do not favor one strategy over another [51, 52]. In view of their demonstrated safety and efficacy against this serious childhood infection, the WHO recommends that Hib conjugated vaccines be included in all routine infant immunization programs [53]. As of 2018, 191 countries had introduced Hib vaccines into their childhood vaccine programs. Global coverage with 3 doses of Hib vaccine is estimated at 72%, with great variation between regions. The WHO regions of the Americas and South East Asia are estimated to have 87% coverage, whereas the WHO Western Pacific Region has only 23% [54], driven by the poor awareness of the infection in China.

In the United States during 2017, >15.3 million children received the Hib vaccine primary series by age 24 months. Coverage averaged 92.3% and varied by state, ranging from 87.4% (Oregon) to 97.1% (Virginia) [55].

## IMPACT OF HIB IMMUNIZATION ON HIB DISEASE

Hib causes terrible infections in young children that carry high morbidity and mortality, and Hib vaccines have dramatically reduced both. Impact studies of Hib vaccines generally target Hib invasive infections—defined as an illness compatible with an invasive disease such as meningitis or sepsis with isolation of the bacterium from a normally sterile site—because cases are readily identified, the microbial cause is usually clearly established, and these are the most common Hib diseases. Worldwide, prior to availability of Hib vaccines, meningitis accounted for 52% of Hib infections, pneumonia 12%, epiglottitis 11%, and others such as cellulitis, septic arthritis, and bacteremia without a focus 25% [56]. The estimated annual toll of Hib disease in children 0–4 years of age was 83 × 10^6^ in developed countries and 550 × 10^6^ in developing countries, with the case fatality rates ranging from 2% to 5% in developed countries and 10% to 30% in developing countries. In older adults and immunocompromised individuals, the mortality rate for Hib disease is approximately 25% [57].

Reduction of Hib invasive infection following childhood Hib immunization results from both the direct protective effect of vaccination (ie, vaccine-induced immunity in the vaccine recipient) as well as the indirect protective effect of herd immunity, which results from vaccine-induced reduction in pharyngeal Hib colonization in vaccinees and, thus, reduced community spread. Four years following introduction of Hib conjugate vaccine in Finland, 3.5% of unvaccinated children carried Hib in their oropharynges compared to 0% of vaccinated children [58]. Similarly, in the United Kingdom, unvaccinated siblings within families of vaccinated children acquired Hib colonization less readily than siblings of unvaccinated controls [59]. Shortly after the introduction of HbOC in Los Angeles, Hib disease incidence dropped significantly, from 24.2 to 4.4/100 000, even though the vaccine coverage was only 20%–60%, suggesting protection beyond direct receipt of the vaccine, likely due to decreased carriage among vaccinated children [43]. In The Gambia, because many children were vaccinated after the age of highest incidence, Adegbola et al estimated that 49% of Hib prevention resulted from indirect effects [60], such as reduction in carriage among vaccinated children.

### Impact of Hib Vaccine in North America

**Figure 1.**
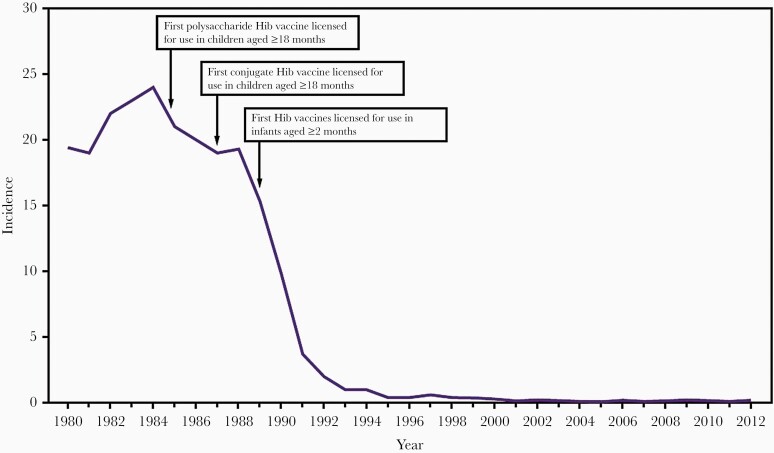
Impact of *Haemophilus influenzae* type b (Hib) vaccines on incidence per 100 000 children <5 years old in the United States, 1980–2012 [29].

Societal benefits from Hib immunization were dramatic and appeared very quickly after vaccine initiation. After introduction of HbOC and PRP-OMP vaccines to 2-month-old infants in the United States in 1991, the incidence of Hib infection declined dramatically (Figure 1). In Southern California, Hib disease in children 0–5 years old decreased 95% in 3 years [61] and in Minnesota and in Dallas, Texas, the incidence of Hib infection decreased >80% in the 3 years after the switch from PRP vaccines to conjugate Hib vaccines [62].

**Figure 2.**
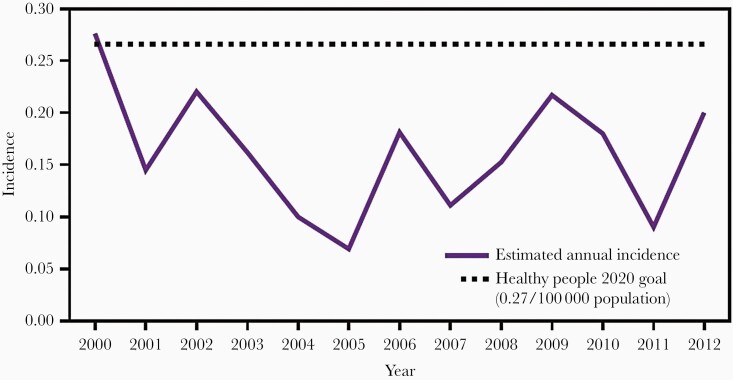
Estimated annual incidence per 100 000 children aged <5 years of *Haemophilus influenzae* type b infection in the United States, 2000–2012 [29].

Since 1991, Hib infection has been a nationally reported disease in the United States, and the Centers for Disease Control and Prevention (CDC) has monitored Hib disease through reports from the state health departments and the Active Bacterial Core surveillance system in several sites [63]. In 2000, approximately 80% of the *H. influenzae* isolates were serotyped and vaccine coverage was >90% [64]. Based on data from these sources, after 2000, the rates of Hib invasive infections in all ages had fallen to <0.3/100 000 (Figure 2), a decrease of 99% compared to 23/100 000 during the prevaccine years. By state, excluding Alaska, average annual incidence rates in 1998–2000 ranged from 0 to 2.1/100 000; the rate in Alaska was 9.4/100 000 [63] possibly because of crowded households and high colonization rates resulting from the lower antibody levels generated by PRP-OMP [25].

An analysis from the CDC including the years 1989–2008 showed the estimated annual incidence of Hib invasive infection in the United States had fallen to 0.05 cases/100 000 in all ages; in adults aged >65 years the incidence was 0.11/100 000 and in children <1 year of age 0.71/100 000. In children 1–4 years of age, incidence was 0.11/100 000 [65]. By 2009–2015, the incidence for all age groups had further fallen to 0.03/100 000, with the highest rates in adults ≥50 years (0.04/100 000) and in children <1 year of age (0.30/100 000) [66]. In children 1–4 years of age, incidence had fallen to 0.08/100 000. As of 2017, the CDC estimated 0.19 cases/100 000 children <5 years of age, fulfilling its Healthy People 2020 goal of <0.27/100 000 [67].

Thus, the incidence of invasive Hib disease has decreased in all ages in the United States, likely due to strong vaccine coverage and herd immunity related to the vaccine’s ability to reduce Hib pharyngeal colonization. The WHO estimates Hib vaccine coverage in 2019 in the United States to be 92% [54].

In Canada, the WHO estimates Hib vaccine coverage in 2019 to be 91%, a decrease from the peak of 98% in 1998 [54]. Similar to the United States, success of conjugated Hib vaccines has resulted in <2% of prevaccine disease totals in Canadian children [68]. Furthermore, Hib disease in unvaccinated adults was reduced in Ontario, likely the result of decreased Hib carriage in vaccinated children [69].

### Impact of Hib Vaccine in Europe and Australia

Forty-three (79%) of WHO member countries in the European region conduct Hib disease surveillance with laboratory confirmation [70], and in 2019 only 6 member nations reported <90% Hib vaccine coverage: Montenegro (86%), Romania (86%), Austria (85%), Belarus (76%), Bosnia and Herzegovina (62%), and Ukraine (58%) [54].

For unknown reasons, prior to the introduction of Hib vaccines, the annual incidence of Hib meningitis in children <5 years old in Europe was half that in the United States (23/100 000 vs 50–60/100 000) [56], with fairly high regional variation. Nevertheless, the reduction in the incidence of Hib meningitis and other invasive diseases following introduction of the Hib conjugate vaccines in Europe was, as in the United States, swift and large. For example, 2 years after initiating PRP-T, the incidence of Hib meningitis declined 87% in the North East Thames region of the United Kingdom [71]. Furthermore, 4 years after introduction of Hib conjugate vaccine in the Rhein-Main region of Germany, incidence of Hib infection in children had declined by 97% [72]. Similarly, dramatic declines were seen in Iceland [73] and France [74], as well as the Basque region of Spain, Austria, Ireland, and Australia [56]. Sustained effectiveness of Hib vaccines in Europe continued after the widespread use of combination vaccines [75].

An Hib surveillance study sponsored by WHO in Moscow determined the annual incidence of Hib meningitis to be 5.7/100 000 [76], and an economic analysis of the use of Hib vaccine in 2006 concluded that the vaccine in the Russian Federation national vaccine program was not cost-effective because of the high price of the vaccine. Nevertheless, by 2012 the Hib vaccine was included in the national vaccine program for “high risk” children using 3 doses in the primary series and a booster dose [77, 78], and the uptake in that year (the only year recorded in the WHO database) was said to be 90% [79]. A newly licensed pentavalent vaccine containing a Hib conjugate antigen manufactured in Russia may increase Hib vaccine use to all Russian children [80].

A government-funded Hib vaccine program was introduced in Australia in May 1993, and by August the vaccination rates had risen from 9% to 43% [81]. As a result of this program using PRP-T, Hib disease incidence rates for all ages halved from 0.13/100 000 in 2000 to 0.06 in 2017 [82]. Of the 345 cases reported, 33% occurred among Aboriginal and Torres Strait Islander peoples and, although incidence rates of disease among Indigenous Australians declined during the 17-year study, they still remained approximately 8 times higher than those of non-Indigenous Australians, likely because Hib infection occurs at younger ages, and complete Hib immunization occurs at older ages in Indigenous children compared with the non-Indigenous.

### Impact of Hib Vaccine in Resource-Limited Countries

Hib conjugate vaccines have been introduced in 191 countries worldwide, and the WHO estimates the global coverage to be 72% (the highest recorded), with wide regional variation [54]. In 2019, 6 member nations failed to achieve >60% coverage: Venezuela (60%), Ukraine (58%), Nigeria (58%), Papua New Guinea (50%), South Sudan (49%), and Samoa (44%).

Barriers to Hib vaccination in resource-limited countries include uncertainty of the need for the vaccine because of poor data on disease burden and poor recognition of the vaccine’s potential impact; difficulty in conducting surveillance studies; the relatively high cost of the vaccine; and the inability to coordinate vaccination campaigns [83].

In addition, barriers to accurate assessment of vaccine impact include limited high-quality, population-based surveillance systems that permit calculation of disease burden, both pre- and postintroduction of vaccines [84]. In 2005 Gavi, the Vaccine Alliance, committed US$37 million to fund the Hib initiative [85]. Since the inception of this program, the cost of Hib vaccines supplied by Gavi has steadily decreased such that the 2018 price of DTwP-HepB-Hib is US$0.87 per dose, a 70% decline since 2014.

With funding assistance from the pharmaceutical industry, the government of The Gambia initiated a PRP-T vaccine initiative in 1997, and funding was assumed by Gavi in 2002. By 2007, the annual incidence of Hib meningitis in children under age 5 years in The Gambia dropped from 60/100.000 to 0 despite irregular vaccine supply, and the Hib colonization rate dropped from 12% to 0.25% [60]. Six years later, in 2013, the annual incidence of Hib meningitis in children <5 years of age and the Hib colonization rate remained low (1.3/100 000 and 0.9%, respectively) [86].

Similar dramatic decreases in Hib meningitis have been seen in other Gavi-supported immunization programs in Africa that implemented PRP-T, including Kenya [87], Uganda [88], Malawi [89], Senegal [90], and Rwanda [91], despite a vaccine schedule of 3 doses in the primary series and no booster. After the initial dramatic decrease in Hib invasive disease in South Africa, incidence rates rose from 0.7/100 000 population in 2003 to 1.3/100 000 in 2009; half of the increase represented vaccine failures, which were seen in both HIV-infected and noninfected children [92]. In the Grand Casa Prefecture of Morocco, a middle-income country, the annual incidence of Hib meningitis decreased by 93%, from 15/100 000 to 1/100 000, in the 3 years following introduction of 3 doses of Hib vaccine [93].

Yet, many African children remain unvaccinated. A recent study from the Gavi target country Ethiopia, with Hib vaccine coverage of only 53.2%, illustrates factors strongly associated with inadequate immunization: rural residency, household poverty, traditional religion, ethnicity, reduced maternal education, and female head of household [94].

Until recently, the Hib disease burden in Asia was thought to be lower than other areas of the world, possibly due to use of antibiotics prior to blood and/or cerebrospinal fluid collection or suboptimal surveillance systems, and, thus, the benefits of Hib vaccines were underappreciated [95, 96]. Mongolia was one of the first Asian countries to introduce the Hib conjugate vaccine, and the incidence of Hib meningitis in Ulaanbataar fell from 28/100 000 children in 2002–2005 to 2/100 000 in 2008–2010 [97]. In Pakistan, introduction of the conjugate Hib vaccine reduced Hib meningitis by 89% and 93% compared to community and hospital controls, respectively [98]. In Singapore, following introduction of Hib vaccine in 2004, Hib invasive disease fell to an annual incidence of 0.57/100 000, a reduction of 86.4%; from 2008 to 2012, the annual incidence fell further to 0.2/100 000, a reduction of 95% [99]. After an economic analysis in 2012 demonstrated it to be cost-effective [100], Hib vaccine was introduced into the national immunization program of India in 2013 as the pentavalent DPT, HepB, PRP-T vaccine, and by 2019, coverage was 97% [79]. In China, where Hib carriage is low [101], Hib vaccine is not included in the national vaccine program, and Hib immunization requires out-of-pocket payment; thus, Hib vaccine coverage in China is only 45% [102].

### Impact of Hib Vaccine on Pneumonia

Diagnosis of Hib pneumonia is most secure in cases with concomitant Hib bacteremia, as establishing the causative organism of nonbacteremic pneumonia requires invasive procedures such as lung taps or deep tracheal aspirates. Furthermore, serotyping is often not reported for *H. influenzae* isolated from patients with pneumonia. Prior to the availability of the Hib vaccine, the incidence of Hib pneumonia in children 0–4 years of age was estimated to be 6/100 000 in developed countries and 300/100 000 in developing countries, with a case fatality rate of 5% and 13%–24%, respectively [56].

Studies using case-control methodology demonstrated Hib vaccine effectiveness against pneumonia in children primarily <12 months of age to be 62%–70% in Pakistan [103] and in children <25 months of age to be 56% in Ukraine [104]. A meta-analysis of 6 papers from developing countries [105] showed that receipt of Hib conjugated vaccines by children under age 5 years was estimated to reduce radiographically proven pneumonia by 18%, clinically diagnosed severe pneumonia by 6%, and all clinically diagnosed pneumonia by 4%. Similar reductions in Hib pneumonia in children were seen in Mozambique [106].

### Impact of Hib Vaccine on Epiglottitis

Prior to the availability of the Hib vaccine, the incidence of Hib epiglottitis in children 0–4 years was estimated to be 13/100 000 in developed countries and <1/100 000 in developing countries, with a case fatality rate of 2% and 20%, respectively [56]. With the advent of the Hib vaccine in Finland, the annual incidence among children <5 years of age dropped from 13.2/100 000 in 1985–1986 to 0.3/100 000 in 1992 [107]. Similar decreases in Hib epiglottitis in children was observed in Australia following introduction of the Hib vaccine, with most epiglottitis now caused by other organisms or seen in older children or adults [108].

### Impact of Hib Vaccine on People With Sickle-Cell Disease

Prior to the availability of Hib vaccine, Hib was described in some studies as the most common cause of bacteremia among children with sickle-cell disease in the United States [109] as well as in African countries, where Hib bacteremia was 13 times more common among sickle-cell disease patients than among controls [110]. Hib disease burden among those with sickle-cell disease in high-resource countries has dramatically decreased with widespread use of Hib conjugate vaccines [111]; over a 7-year period, no cases of Hib bacteremia were identified among children with sickle-cell disease in Atlanta, Georgia [112].

### Impact of Hib Vaccine on Mortality

Hib vaccine saves lives. WHO estimates Hib represents 3% of all-cause mortality in children under 5 years of age [113], with 363 000 childhood deaths from Hib in 2000 globally. Among children aged <5 years in low-income countries, receipt of Hib conjugated vaccines was estimated to reduce all-cause mortality by 5% [105]. A meta-analysis of 4 case control studies estimated the protective effect of Hib vaccination on meningitis mortality to be 38%–43% [114]. A Gavi-sponsored study estimates that Hib vaccine averted almost 1.4 million deaths from 2011 to 2000 in 73 Gavi-eligible countries, or 2.6 deaths averted per 1000 persons vaccinated [115].

### Impact of Hib Vaccine on Special Populations

Viral immunosuppression places HIV--infected children at increased risk for bacterial infection, particularly with encapsulated bacteria. Initial antibody responses to PRP-T vaccines were only slightly reduced among HIV-infected infants, but at 1 year postimmunization the antibody titers of 43% of those children were below the level associated with long-term protection [116]. Indeed, HIV-infected children are 20 times more likely to experience bacteremic Hib pneumonia than otherwise healthy children [117]. On the other hand, Hib meningitis occurs significantly less often among HIV-infected children compared with otherwise healthy children [117], but neurologic sequelae are twice as common [118]. Hib vaccine effectiveness against invasive Hib infection was only 44% among fully vaccinated, HIV-infected children in South Africa (only 1 of the 7 vaccine failures had meningitis and 6 had pneumonia) compared to 97% among fully vaccinated, non-HIV-infected children [119]. Following introduction of Hib conjugate vaccine in Mozambique, with relatively high HIV prevalence, 5 children developed Hib bacteremia without meningitis, and 4 of the 5 were HIV positive. The median age of the Hib-infected children was 28 months and 1 had completed the full Hib immunization series [106]. In Malawi, 14% of children with Hib meningitis were also HIV infected, and the effectiveness of 2 or more doses of Hib vaccine in preventing Hib meningitis among HIV-infected children was 100% [89].

The very high incidence of Hib meningitis—474/100 000 children under age 5 years [120]—in Bethel, Alaska, stimulated studies of Hib infection and responses to vaccines among native Alaskans. Early, widespread use of PRP-OMP after 1991 resulted in a decrease in Hib disease of 94% among Alaska natives and 96% among nonnatives, related to the protective immunity PRP-OMP generates with the first dose at age 2 months. A change to HbOC vaccine in 1996 resulted in a rapid increase in annual Hib disease incidence among rural Alaska Natives under age 5 years from 19.8 to 91.1 cases/100 000, because full protection from HbOC occurs only after the 6-month dose, and many Alaska Native children are infected between ages 4 and 6 months. Subsequent to the return to PRP-OMP use in Alaska in 2001, annual incidence rates of Hib disease among children under age 5 years decreased to 5.4 and 0/100 000 among Native and non-Native Alaskans, respectively [45]. Despite widespread vaccine use, Hib colonization among rural Native Alaskan children remains relatively high, which, along with household crowding, may contribute to the overall lower effectiveness of PRP-OMP among Native Alaskans [121].

Prior to introduction of the Hib vaccine among American Indian children living on the Navajo Nation reservation, incidence of Hib disease in children under age 5 years was 214/100 000 [39]; among children on the White Mountain Apache reservation, the incidence of *H. influenzae* meningitis was 264/100 000 (82.3% were serotype b) [122]. Following introduction of the Hib vaccine, the mean rate of Hib infections among Navajo and Whitewater Apache children aged <5 years was 5.8/100 000 from 2000 to 2003 [123], and, as seen in other populations, the decline in Hib diseases was associated with direct as well as indirect protective effects of vaccination [124].

Among Inuit children under age 5 years in northern Manitoba, prior to the introduction of the Hib vaccine, the incidence of Hib meningitis was 530/100 000 [125] compared to the incidence of all Hib infections among Canadian Indigenous children following introduction of the vaccine of 8.3–17/100 000 [126]. Among Indigenous children under age 5 years in Australia, the incidence of Hib infection prevaccine was 278–529/100 000 compared with 4.3/100 000 postvaccine and among Maori children in New Zealand under age 5 years, the comparable rates were 38/100 000 and 4.1/100 000, respectively [126].

Thus, although widespread use of Hib vaccines has resulted in significant reductions in Hib infections in Indigenous children around the world, their rates of Hib disease still remain higher than those of non-Indigenous children in their respective countries.

## ECONOMIC IMPACT OF HIB VACCINES

In 2018, the cost of pentavalent vaccine containing DTwP, PRP-T, and HepB was US$0.87 in 73 Gavi-eligible countries and, among non-Gavi-eligible countries, US$1.08 in 4 Pan American Health Organization (PAHO) countries and US$120–$456 in 17 non-PAHO countries [127]. In the WHO-sponsored, global assessment of the economic impact of Hib vaccine published in 2017, half of the studies included in the analysis were from resource-poor countries [128]. Nearly 85% of the 27 studies demonstrated either cost-saving or cost-effectiveness. The most influential factors in determining cost-effectiveness included rate of Hib infection (which varied widely), vaccine price, discount rate, vaccine efficacy, and vaccine coverage rate. The study from Russia demonstrated that for the Hib vaccine to be cost-effective, the vaccine price had to reduce about 90% of the current price [76]. Similarly, in Thailand, where the cost per dose (US$9.98) accounted for 80% of the overall cost of vaccination, the price would have to decline by 92% to be cost-effective [129]. A Gavi-sponsored study estimated that between 2011 and 2020, Hib immunization has averted US$53.6 billion in cost of illness, with US$6.4 billion in life-year disability averted, and the overall economic and social value of Hib vaccine to be US$820 billion [130].

A goal of Gavi has been to influence the market for pentavalent Hib vaccine, and its strategy over 15 years was successful. During 2001–2018, Gavi disbursed US$3.5 billion to support use of 50 million pentavalent doses annually before 2005, increasing to 300 million doses annually by 2016. By 2018 the vaccine supply allowed 80 million children per year to be immunized, a 16-fold increase from 2005, with vaccine-related costs per child at one-quarter the 2005 level for donors and countries [131].

## IMPACT OF HIB VACCINES ON MICROBIAL ANTIBIOTIC RESISTANCE

In addition to reduced morbidity and mortality from bacterial infections, antibacterial vaccines offer the possibility of reducing antibiotic resistance among organisms that colonize, and potentially infect, vaccine recipients. Indeed, Hib vaccine has been shown to significantly reduce Hib infections in Finland such that Hib resistant to β-lactam antibiotics need not be considered in the differential diagnosis of septic arthritis in immunized, young children [132]. Thus, narrower-spectrum empiric antibiotic therapy may be initiated.

## THE FUTURE OF *H. INFLUENZAE* VACCINES

With the widespread use of Hib vaccines, current *H. influenzae* infections in children in high-resource areas are most commonly serotypes a, f, or nontypeable (because they lack a capsule) [65]. In 2018 in the United States, among 626 cases of *H. influenzae* infections in children under age 5 years reported to the CDC, 38 (6.1%) were serotype b, 222 (35.5%) were nontypeable, 175 (27.9%) were unknown serotype, and 191 (30.5 %) were non-b serotypes [133]. Serotype a (Hia) is predominant among *H. influenzae* invasive infections among American and Canadian indigenous peoples [123, 134–136]. Hib immunization has not been demonstrated to drive serotype replacement among *H. influenzae* encapsulated strains [137].

Although the burden of Hia disease in the United States is too low for meaningful efficacy trials of a vaccine against Hia [138], an analysis from Canada suggests such a vaccine could be cost-effective [139]. A vaccine that contains protein D of nontypeable *H. influenzae*, used as a carrier protein for multivalent pneumococcal capsule vaccines, has been implemented abroad but not licensed in the United States. Postmarketing studies suggest this formulation may make an impact on acute otitis media associated with nontypeable *H. influenzae* [140]. Indeed, a highly effective vaccine against nontypeable *H. influenzae* otitis media would considerably reduce the burden of otitis media, and would significantly lessen the impact of otitis media treatment on antibiotic resistance among colonizing bacteria.

While the availability and widespread use of Hib vaccines have dramatically reduced Hib disease among young children in many parts of the world, additional efforts must be expended to facilitate access to these vaccines for all children, everywhere. Furthermore, complex humanitarian crises threaten vaccine programs in many areas of the world. Indeed, even in the United States, the severe acute respiratory syndrome coronavirus 2 pandemic of 2020 seriously disrupted routine childhood vaccination [141, 142]. Hib has been identified as a vaccine-preventable disease that contributes disproportionately to morbidity and mortality during complex humanitarian crises, and Hib vaccine has been targeted for inclusion in standard emergency response efforts [143].

In summary, Hib vaccines have nearly eliminated the considerable childhood morbidity and mortality associated with *H. influenzae* infections in countries that have implemented widespread Hib vaccination programs.

## Supplementary Data

Supplementary materials are available at *The Journal of Infectious Diseases* online. Consisting of data provided by the authors to benefit the reader, the posted materials are not copyedited and are the sole responsibility of the authors, so questions or comments should be addressed to the corresponding author.

The complete references are available as online Supplemental Material.

## Supplementary Material

jiaa537_suppl_Supplementary-Material

## Notes

***Supplement sponsorship.*** This supplement is sponsored by the Bill and Melinda Gates Foundation.

***Potential conflicts of interest.*** Author certifies no potential conflicts of interest.
