# Supplementary material for: Hib Vaccines: Their Impact on Haemophilus influenzae Type b Disease
Source: J Infect Dis. 2021 Sep 30;224(Suppl 4):S321–30. doi: 10.1093/infdis/jiaa537 (PMC8482018; doi:10.1093/infdis/jiaa537)
Supplement: jiaa537_suppl_Supplementary-Material [file jiaa537_suppl_supplementary-material.docx]

1. Fothergill LD, Wright J. Influenzal meningitis: the relation of age incidence to the bacteridal power of blood against the causal organism. J Immunol. 1933;24(4):273-84.

2. Cochi SL, Broome CV, Hightower AW. Immunization of US children with *Hemophilus influenzae* type b polysaccharide vaccine: A Cost-effectiveness Model of Strategy Assessment. JAMA. 1985;253(4):521-9.

3. Broome CV. Epidemiology of *Haemophilus influenzae* type b infections in the United States. Pediatr Infect Dis J. 1987;6(8):779-82.

4. Peltola H, Aavitsland P, Hansen KG, Jonsdottir KE, Nokleby H, Romanus V. Perspective: A five-country analysis of the impact of four different *Haemophilus influenzae* type b conjugates and vaccination strategies in scandinavia. J Infect Dis. 1999;179(1):223-9.

5. Baraff L, Lee S, Schriger D. Outcomes of bacterial meningitis in children: a meta-analysis. Pediatr Infect Dis J. 1993;12(5):389 - 94.

6. Chandran A, Herbert H, Misurski D, Santosham M. Long-term sequelae of childhood bacterial meningitis. Pediatr Infect Dis J. 2011;30(1):3-6.

7. Wenger JD, Hightower AW, Facklam RR, Gaventa S, Broome CV, The Bacterial Meningitis Study Group. Bacterial meningitis in the united states, 1986: Report of a multistate surveillance study. J Infect Dis. 1990;162(6):1316-23.

8. Schlech WF, III, Ward JI, Band JD, Hightower A, Fraser DW, Broome CV. bacterial meningitis in the United States, 1978 through 1981: The national bacterial meningitis surveillance study. JAMA. 1985;253(12):1749-54.

9. Zamenhof S, Leidy G, Fitzgerald PL, Alexander HE, Chargaff E. Polyribophosphate, the type-specific substance of *Hemophilus influenzae*, type B. J Biol Chem. 1953;203(2):695-704.

10. Tillett WS, Francis T. Serological reactions in pneumonia with a non-protein somatic fraction of pneumococcus. J Exp Med. 1930;52(4):561-71.

11. Gotschlich EC, Goldschneider I, Artenstein MS. Human immunity to the meningococcus. J Exp Med. 1969;129(6):1367-84.

12. Anderson P, Kayhty H, Makela PH. The protective level of serum antibodies to the capsular polysaccharide of *Haemophilus influenzae* type B [with Reply]. J Infect Dis. 1984;149(6):1034-5.

13. Käyhty H, Peltola H, Karanko V, Mäkelä PH. The protective level of serum antibodies to the capsular polysaccharide of *Haemophilus influenzae* Type b. J Infect Dis. 1983;147(6):1100-.

14. Ward JI, Broome CV, Harrison LH, Shinefield H, Black S. *Haemophilus influenzae* type b vaccines: Lessons for the future. Pediatrics. 1988;81(6):886.

15. Peltola H, Käyhty H, Virtanen M, Mäkelä PH. Prevention of *Hemophilus influenzae* type b bacteremic infections with the capsular polysaccharide vaccine. N Engl J Med. 1984;310(24):1561-6.

16. Landsteiner K, van der Scheer J. Serological differentiation of steric isomers. J Exp Med. 1928;48(3):315-20.

17. Avery OT, Goebel WF. Chemo-immunological studies on conjugated carbohydrate-proteins. J Exp Med 1931;54(3):437-47.

18. Schneerson R, Barrera O, Sutton A, Robbins JB. Preparation, characterization, and immunogenicity of *Haemophilus influenzae* type b polysaccharide-protein conjugates. J Exp Med. 1980;152(2):361-76.

19. Anderson P. Antibody responses to *Haemophilus influenzae* type b and diphtheria toxin induced by conjugates of oligosaccharides of the type b capsule with the nontoxic protein CRM_197_. Infection and Immunity. 1983;39(1):233-8.

20. Marburg S, Jorn D, Tolman RL, et al. Bimolecular chemistry of macromolecules: synthesis of bacterial polysaccharide conjugates with Neisseria meningitidis membrane protein. J Am Chem Soc. 1986;108(17):5282-7.

21. Paul W, Kung J, Ahmed A, Stein K. B lymphocyte subpopu!ations and responses to polysaccharide antigens. In: Sell SH, Paul WF, editors. *Haemophilus influenzae*: epidemiology, immunology, and prevention of disease. New York: Elsevier Science; 1982. p. 121-8.

22. Weinberg GA, Granoff DM. Polysaccharide-protein conjugate vaccines for the prevention of *Haemophilus influenzae* type b disease. J Pediatr. 1988;113(4):621-31.

23. Eskola J, Peltola H, Takala AK, et al. Efficacy of *Haemophilus influenzae* Type b Polysaccharide–Diphtheria toxoid conjugate vaccine in infancy. N Engl J Med. 1987;317(12):717-22.

24. Eskola J KH, Takala AK, Peltola H, et al. A randomized, prospective field trial of a conjugate vaccine in the protection of infants and young children against invasive *Haemophilus influenzae* type b disease. N Engl J Med. 1990;323(20):1381 - 7.

25. Decker MD, Edwards KM, Bradley R, Palmer P. Comparative trial in infants of four conjugate *Haemophilus influenzae* type b vaccines. J Pediatr. 1992;120(2):184-9.

26. Takala A EJ, Peltola H, Makela PH. Epidemiology of invasive *Haemophilus*

*influenzae* type b disease among children in Finland before vaccination with *Haemophilus*

*influenzae* type b conjugate vaccine. Pediatr Infect Dis J. 1989;8:297-302.

27. Black S, Shinefield H, Fireman B, Hiatt R, Polen M, Vittinghoff E. Efficacy in infancy of oligosaccharide conjugate *Haemophilus influenzae* type b (HbOC) vaccine in a United States population of 61 080 children. Pediatr Infect Dis J. 1991;10(2):97-104.

28. Shapiro ED, Capobianco LA, Berg AT, Zitt MQ. The immunogenicity of *Hemophilus influenzae* type B polysaccharide-*Neisseria meningitidis* Group B outer membrane protein complex vaccine in infants and young children. J Infect Dis. 1989;160(6):1064-7.

29. Briere EC, Rubin L, Moro PL, Cohn A, Clark T, Messonnier N. Prevention and control of *Haemophilus influenzae* type b disease: Recommendations of the Advisory Committee on Immunization Practices (ACIP). MMWR Recommendations & Reports. 2014;63(1):1-14.

30. Briere EC. Food and drug administration approval for use of hiberix as a 3-dose primary *Haemophilus influenzae* type b (Hib) vaccination series. MMWR Morbidity and Mortality Weekly Report. 2016;65(16):418 – 9.

31. Pichichero ME. Protein carriers of conjugate vaccines: characteristics, development, and clinical trials. Hum Vaccin Immunother. 2013;9(12):2505-23.

32. D'Angio CT, Murray TE, Li L, et al. Immunogenicity of *Haemophilus influenzae* type b protein conjugate vaccines in very low birth weight infants. Pediatr Infect Dis J. 2013;32(12):1400-2.

33. Granoff DM, Anderson EL, Osterholm MT, et al. Differences in the immunogenicity of three *Haemophilus influenzae*  type b conjugate vaccines in infants. J Pediatr. 1992;121(2):187-94.

34. Bulkow LR WR, Letson GW, Chang SJ, Ward JI. Comparative immunogenicity of four *Haemophilus influenzae* type b conjugate vaccines in Alaska Native infants. Pediatr Infect Dis J. 1993;12(6):484–91.

35. Galil K, Singleton R, Levine OS, et al. Reemergence of invasive *Haemophilus influenzae* type b disease in a well-vaccinated population in remote alaska. J Infect Dis. 1999;179(1):101-6.

36. Santosham M, Wolff M, Reid R, et al. The efficacy in Navajo Infants of a conjugate vaccine consisting of *Haemophilus influenzae* type b polysaccharide and *Neisseria meningitidis* outer-membrane protein complex. N Engl J Med. 1991;324(25):1767-72.

37. Ward JI, Lum MKW, Hall DB, Silimperi DR, Bender TR. Invasive *Haemophilus influenzae* type b disease in Alaska: background epidemiology for a vaccine efficacy trial. J Infect Dis. 1986;153(1):17-26.

38. Ward JI, Lum MW, Margolis HS, Fraser DW, Bender TR, Anderson P. *Haemophilus influenzae* disease in Alaskan Eskimos: Characteristics of a population with an unusual incidence of invasive disease. Lancet. 1981;317(8233):1281-5.

39. Coulehan JL, Richard HM, Christian H, Richard S, Thomas KW, Joseph SCK. Epidemiology of *Haemophilus influenzae* type B disease among Navajo Indians. Public Health Rep. 1984;99(4):404-9.

40. Guerra FA, Blatter MM, Greenberg DP, Pichichero M, Noriega FR. Safety and immunogenicity of a pentavalent vaccine compared with separate administration of licensed equivalent vaccines in US infants and toddlers and persistence of antibodies before a preschool booster dose: A randomized, clinical trial. Pediatrics. 2009;123(1):301.

41. Obando-Pacheco P, Rivero-Calle I, Gómez-Rial J, Rodríguez-Tenreiro Sánchez C, Martinón-Torres F. New perspectives for hexavalent vaccines. Vaccine. 2018;36(36):5485-94.

42. Shapiro ED, Ward JI. The epidemiology and prevention of disease caused by *Haemophilus influenzae* type b. Epidemiol Rev. 1991;13(1):113-42.

43. Vadheim CM, Greenberg, DP, Eriksen, E, Hemenway, L., et al. Protection provided by *Haemophilus influenzae* type b conjugate vaccines in Los Angeles County. Pediatr Infect Dis J. 1994;13(4):274–80.

44. Booy R, Hodgson S, Moxon ER, et al. Efficacy of *Haemophilus influenzae* type b conjugate vaccine PRP-T. Lancet. 1994;344(8919):362-6.

45. Singleton R, Hammitt L, Hennessy T, et al. The Alaska *Haemophilus influenzae* type b experience: lessons in controlling a vaccine-preventable disease. Pediatrics. 2006;118(2):e421-9.

46. Ward JI, Brenneman G, Letson GW, Heyward WL. Limited efficacy of a *Haemophilus influenzae* type b conjugate vaccine in Alaska Native infants. The Alaska *H. influenzae* Vaccine Study Group. N Engl J Med. 1990 323(20):1393-401.

47. Heath PT, Booy R, Griffiths H, et al. Clinical and Immunological risk factors associated with *Haemophilus influenzae* type b conjugate vaccine failure in childhood. Clin Infect Dis. 2000;31(4):973-80.

48. Lee YC, Kelly DF, Yu L-M, et al. *Haemophilus influenzae* type b vaccine failure in children is associated with inadequate production of high-quality antibody. Clin Infect Dis. 2008;46(2):186-92.

49. CDC. VAERS database Center for Disease Control and Prevention 2020. Available from: https://wonder.cdc.gov/controller/datarequest/D8;jsessionid=ABC1066AC8CBBAFB7E08BC499CE9DD54. Accessed June 2, 2020.

50. Vaccine Safety Committee, Institute of Medicine. Adverse events associated with childhood vaccines: evidence bearing on casuality. Stratton KR, Howe CJ, Johnston J, Richard B., editors. Washington DC: National Academy Press; 1994.

51. Jackson CMA, Mangtani P, Fine P. Effectiveness of *Haemophilus influenzae* type b vaccines administered according to various schedules: systematic review and meta-analysis of observational data. Pediatr Infect Dis J 2013;32:1261.

52. Low N, Redmond, SM, Rutjes, AS, et al. Comparing *Haemophilus influenzae* type b conjugate vaccine schedules: a systematic review and meta-analysis of vaccine trials. Pediatr Infect Dis J 2013;32:1245.

53. WHO. *Haemophilus influenzae* type b (Hib): World Health Organization 2014. Available from: https://www.who.int/immunization/diseases/hib/en/. Accessed June 2, 2020.

54. WHO. Global immunization coverage 2018: WHO; 2019 Available from: https://www.who.int/en/news-room/fact-sheets/detail/immunization-coverage. Accessed June 3, 2020.

55. CDC. *Haemophilus influenzae* type b (Hib) vaccination coverage among children 19-35 months by State, HHS Region, and the United States, National Immunization Survey-Child (NIS-Child), 1995 through 2017: Center for Disease Control and Prevention 2017. Available from: https://www.cdc.gov/vaccines/imz-managers/coverage/childvaxview/data-reports/hib/trend/index.html. Accessed June 1, 2020.

56. Peltola H. Worldwide *Haemophilus influenzae* type b disease at the beginning of the 21st century: global analysis of the disease burden 25 years after the use of the polysaccharide vaccine and a decade after the advent of conjugates. Clin Microbiol Rev. 2000;13(2):302-17.

57. Takala AK, Eskola J, van Alphen L. Spectrum of invasive *Haemophilus influenzae* type b disease in adults. Arch Intern Med. 1990;150(12):2573-6.

58. Takala AK, Eskola J, Leinonen M, et al. Reduction of oropharyngeal carriage of *Haemophilus influenzae* type b (Hib) in children immunized with an Hib conjugate vaccine. J Infect Dis. 1991;164(5):982-6.

59. Barbour ML, Mayon-White RT, Coles C, Crook DWM, Moxon ER. The impact of conjugate vaccine on carriage of *Haemophilus influenzae* type b. J Infect Dis. 1995;171(1):93-8.

60. Adegbola RA, Secka O, Lahai G, et al. Elimination of *Haemophilus influenzae* type b (Hib) disease from The Gambia after the introduction of routine immunisation with a Hib conjugate vaccine: a prospective study. Lancet. 2005;366(9480):144-50.

61. Vadheim CM, Greenberg DP, Eriksen E, et al. Eradication of *Haemophilus influenzae* Type b disease in Southern California. Arch Pediatr Adolesc Med. 1994;148(1):51-6.

62. Murphy TV, White KE, Pastor P, et al. Declining incidence of *Haemophilus influenzae* type b disease since introduction of vaccination. JAMA. 1993;269(2):246-8.

63. Bath S, Bisgard K, Murphy T, Shutt K, Rosenstein N. Progress toward elimination of *Haemophilus influenzae* type b invasive disease among infants and children--United States, 1998-2000. MMWR Morbidity and Mortality Weekly Report. 2002;51(11):234.

64. CDC. National, state, and urban area vaccination coverage levels among children aged 19-35 months--United States, 2000. MMWR Morbidity and Mortality Weekly Report. 2001 50(30):637 - 41.

65. MacNeil JR, Cohn AC, Farley M, et al. Current Epidemiology and trends in invasive *Haemophilus influenzae* disease—United States, 1989–2008. Clin Infect Dis. 2011;53(12):1230-6.

66. Soeters HM, Blain A, Pondo T, et al. Current epidemiology and trends in invasive *Haemophilus influenzae* disease-United States, 2009-2015. Clin Infect Dis. 2018;67(6):881-9.

67. CDC. Active Bacterial Core Surveillance Report, Emerging Infections Program Network, *Haemophilus influenzae* 2017. Centers for Disease Control and Prevention 2017. Available from: https://www.cdc.gov/abcs/reports-findings/survreports/hib17.html Accessed June 1, 2020.

68. Scheifele DW, Bettinger JA, Halperin SA, Law B, Bortolussi R. Ongoing control of *Haemophilus influenzae* type B infections in Canadian children, 2004–2007. Ped Infect Dis J. 2008;27(8):755-7.

69. Adam HJ, Richardson SE, Jamieson FB, Rawte P, Low DE, Fisman DN. Changing epidemiology of invasive *Haemophilus influenzae* in Ontario, Canada: Evidence for herd effects and strain replacement due to Hib vaccination. Vaccine. 2010;28(24):4073-8.

70. WHO. Routine immunization profile WHO European Region. Geneva, Switzerland WHO; 2019. Available from: http://www.euro.who.int/__data/assets/pdf_file/0010/420967/WHO-Regional-profile.pdf?ua=1. Accessed June 4, 2020.

71. Urwin G, Yuan MF, Feldman RA. Prospective study of bacterial meningitis in North East Thames region, 1991-3, during introduction of *Haemophilus influenzae* vaccine. BMJ. 1994;309(6966):1412-4.

72. Zielen S, Ahrens P, Hofmann D, et al. Efficacy of Hib vaccine. Lancet. 1994;344(8925):828-9.

73. Jonsdottir K, Hansen H AV, Laxdal T, Stefansson M. Immunization against *Haemophilus influenzae* type b in Iceland. Results after six years use of PRP-D (ProHIBiT(R). Laeknabladid. 1996;82(1):32-8.

74. Reinert P, Liwartowski A, Dabernat H, Guyot C, Boucher J, Carrere C. Epidemiology of *Haemophilus influenzae* type b disease in France. Vaccine. 1993;11:S38-S42.

75. Wang S, Tafalla M, Hanssens L, Dolhain J. A review of *Haemophilus influenzae* disease in Europe from 2000–2014: challenges, successes and the contribution of hexavalent combination vaccines. Expert Rev Vaccines. 2017;16(11):1095-105.

76. Platonov AE, Griffiths UK, Voeykova MV, et al. Economic evaluation of *Haemophilus influenzae* type b vaccination in Moscow, Russian Federation. Vaccine. 2006;24(13):2367-76.

77. Romanenko V, Osipova I, Galustyan A, et al. Immunogenicity and safety of a combined DTPa-IPV/Hib vaccine administered as a three-dose primary vaccination course and a booster dose in healthy children in Russia: a phase III, non-randomized, open-label study. Hum Vaccin Immunother. 2020:1-9.

78. WHO. WHO vaccine-preventable diseases: monitoring system. 2019 global summary: Russian Federation 2019 Available from: https://apps.who.int/immunization_monitoring/globalsummary/schedules?sc%5Bc%5D%5B%5D=RUS&sc%5Bd%5D=&sc%5Bv%5D%5B%5D=HIB&sc%5BOK%5D=OK. Accessed June3, 2020.

79. WHO. Third dose of *Haemophilus influenzae* type B vaccine. Geneva, Switzerland: WHO; 2019. Available from: https://apps.who.int/immunization_monitoring/globalsummary/timeseries/tscoveragehib3.html. Accessed June 5, 2020.

80. RT. Russia registers its first domestically-produced 5-in-1 vaccine Moscow, Russian Federation 2019. [updated April 18] Available from: https://www.rt.com/russia/456938-russia-first-combination-vaccine/. Accessed June 1, 2020.

81. McIntyre PB, Chey T, Smith WT. The impact of vaccination against invasive *Haemophilus influenzae* type b disease in the Sydney region. Med J Aust. 1995;162(5):245-8.

82. Maguire J, Beard F, Méder K, Dey A, Macartney K, McIntyre P. Australian vaccine preventable disease epidemiological review series: invasive *Haemophilus influenzae* type b disease, 2000-2017. Commu Dis Intell 2000;44:1 - 30.

83. Hajjeh R, Mulholland K, Schuchat A, Santosham M. Progress towards demonstrating the impact of *Haemophilus influenzae* type b conjugate vaccines globally. J Pediatr. 2013;163(1 Suppl):S1-S3.

84. O’Loughlin RE, Edmond K, Mangtani P, et al. Methodology and measurement of the effectiveness of *Haemophilus influenzae* type b vaccine: Systematic review. Vaccine. 2010;28(38):6128-36.

85. GAVI. Hib Initiative: a GAVI success story. 2011. https://www.gavi.org/news/media-room/hib-initiative-gavi-success-story. Accessed June 4, 2020.

86. Howie SR, Oluwalana C, Secka O, et al. The effectiveness of conjugate *Haemophilus influenzae* type B vaccine in The Gambia 14 years after introduction. Clin Infect Dis. 2013;57(11):1527-34.

87. Hammitt LL, Crane RJ, Karani A, et al. Effect of *Haemophilus influenzae* type b vaccination without a booster dose on invasive *H. influenzae* type b disease, nasopharyngeal carriage, and population immunity in Kilifi, Kenya: a 15-year regional surveillance study. Lancet Global Health. 2016;4(3):e185-94.

88. Lee EH, Lewis RF, Makumbi I, et al. *Haemophilus influenzae* type b conjugate vaccine is highly effective in the Ugandan routine immunization program: a case-control study. Trop Med Int Health. 2008;13(4):495-502.

89. Daza P, Banda R, Misoya K, et al. The impact of routine infant immunization with *Haemophilus influenzae* type b conjugate vaccine in Malawi, a country with high human immunodeficiency virus prevalence. Vaccine. 2006;24(37-39):6232-9.

90. Cissé MF, Breugelmans JG, Bâ M, et al. The elimination of *Haemophilus influenzae* type b meningitis following conjugate vaccine introduction in Senegal. Pediatr Infect Dis J. 2010;29(6):499-503.

91. Muganga N, Uwimana J, Fidele N, et al. *Haemophilus influenzae* type b conjugate vaccine impact against purulent meningitis in Rwanda. Vaccine. 2007;25(39-40):7001-5.

92. von Gottberg A, Cohen C, Whitelaw A, et al. Invasive disease due to *Haemophilus influenzae* serotype b ten years after routine vaccination, South Africa, 2003-2009. Vaccine. 2012;30(3):565-71.

93. Braikat M, Barkia A, El Mdaghri N, Rainey JJ, Cohen AL, Teleb N. Vaccination with *Haemophilus influenzae* type b conjugate vaccine reduces bacterial meningitis in Morocco. Vaccine. 2012;30(15):2594-9.

94. Geweniger A, Abbas KM. Childhood vaccination coverage and equity impact in Ethiopia by socioeconomic, geographic, maternal, and child characteristics. Vaccine. 2020;38(20):3627-38.

95. Bröker M. Burden of invasive disease caused by *Haemophilus influenzae* type b in Asia. Jpn J Infect Diseases. 2009;62(2):87-92.

96. Peltola H. Spectrum and burden of severe *Haemophilus influenzae* type b diseases in Asia. Bull World Health Organ. 1999;77(11):878-87.

97. Scott S, Altanseseg D, Sodbayer D, et al. Impact of *Haemophilus influenzae* type b conjugate vaccine in Mongolia: Prospective population-based surveillance, 2002-2010. J Pediatr. 2013;163(1):S8-S11.

98. Baqui AH, El Arifeen S, Saha SK, et al. Effectiveness of *Haemophilus influenzae* type B conjugate vaccine on prevention of pneumonia and meningitis in Bangladeshi children: a case-control study. Pediatr Infect Dis J. 2007;26(7):565-71.

99. Thoon KC, Tee NW, Chew L, Chong CY. Near disappearance of childhood invasive *Haemophilus influenzae* type b disease in Singapore. Vaccine. 2014;32(44):5862-5.

100. Gupta M, Prinja S, Kumar R, Kaur M. Cost-effectiveness of *Haemophilus influenzae* type b (Hib) vaccine introduction in the universal immunization schedule in Haryana State, India. Health Policy Plan. 2012;28(1):51-61.

101. Yang Y, Pan X, Cheng W, et al. *Haemophilus influenzae* type b carriage and burden of its related diseases in Chinese children: Systematic review and meta-analysis. Vaccine. 2017;35(46):6275-82.

102. Zheng Y, Rodewald L, Yang J, et al. The landscape of vaccines in China: history, classification, supply, and price. BMC Infect Dis. 2018;18(1):502.

103. Khowaja AR, Mohiuddin S, Cohen AL, et al. Effectiveness of *Haemophilus influenzae* type b conjugate vaccine on radiologically-confirmed pneumonia in young children in Pakistan. J Pediatr. 2013;163(1):S79-S85.

104. Pilishvili T, Chernyshova L, Bondarenko A, et al. Evaluation of the effectiveness of *Haemophilus influenzae* type b conjugate vaccine introduction against radiologically-confirmed hospitalized pneumonia in young children in Ukraine. J Pediatr. 2013;163(1):S12-S8.

105. Theodoratou E, Johnson S, Jhass A, et al. The effect of *Haemophilus influenzae* type b and pneumococcal conjugate vaccines on childhood pneumonia incidence, severe morbidity and mortality. Int J Epidemiol. 2010;39 (Suppl 1):i172-i85.

106. Sigaúque B, Vubil D, Sozinho A, et al. *Haemophilus influenzae* type b disease among children in rural Mozambique: impact of vaccine introduction. J Pediatr. 2013;163(1 Suppl):S19-24.

107. Takala AK, Peltola H, Eskola J. Disappearance of epiglottitis during large-scale vaccination with *Haemophilus influenzae* type B conjugate vaccine among children in Finland. Laryngoscope. 1994;104(6 Pt 1):731-5.

108. Wood N, Menzies R, McIntyre P. Epiglottitis in Sydney before and after the introduction of vaccination against *Haemophilus influenzae* type b disease. Int Med J. 2005;35(9):530-5.

109. Ward J, Smith AL. *Hemophilus influenzae* bacteremia in children with sickle cell disease. J Pediatr. 1976;88(2):261-3.

110. Ramakrishnan M, Moïsi JC, Klugman KP, et al. Increased risk of invasive bacterial infections in African people with sickle-cell disease: a systematic review and meta-analysis. Lancet Infect Dis. 2010;10(5):329-37.

111. Allali S, Chalumeau M, Launay O, Ballas SK, de Montalembert M. Conjugate *Haemophilus influenzae* type b vaccines for sickle cell disease. Cochrane Database Syst Rev. 2018;8(8):CD011199.

112. Yee ME, Bakshi N, Graciaa SH, et al. Incidence of invasive *Haemophilus influenzae* infections in children with sickle cell disease. Pediatr Blood Cancer. 2019;66(6):e27642.

113. WHO. Estimated Hib and pneumococcal deaths for children under 5 years of age, 2000 Geneva, Switzerland WHO; 2009 [Available from: https://www.who.int/immunization/monitoring_surveillance/burden/estimates/Pneumo_hib_2000/en/index1.html. Accessed June 2, 2020.

114. Davis S, Feikin D, Johnson HL. The effect of *Haemophilus influenzae* type B and pneumococcal conjugate vaccines on childhood meningitis mortality: a systematic review. BMC Public Health. 2013;13(3):S21.

115. Lee LA, Franzel L, Atwell J, et al. The estimated mortality impact of vaccinations forecast to be administered during 2011–2020 in 73 countries supported by the GAVI Alliance. Vaccine. 2013;31:B61-B72.

116. Gibb D, Giacomelli A, Masters J, et al. Persistence of antibody responses to *Haemophilus influenzae* type b polysaccharide conjugate vaccine in children with vertically acquired human immunodeficiency virus infection. Pediatr Infect Dis J. 1996;15(12):1097-101.

117. Madhi SA, Petersen K, Madhi A, Khoosal M, Klugman KP. Increased disease burden and antibiotic resistance of bacteria causing severe community-acquired lower respiratory tract infections in human immunodeficiency virus type 1-infected children. Clin Infect Dis. 2000;31(1):170-6.

118. Madhi SA, Madhi A, Petersen K, Khoosal M, Klugman KP. Impact of human immunodeficiency virus type 1 infection on the epidemiology and outcome of bacterial meningitis in South African children. Inter J Infect Dis. 2001;5(3):119-25.

119. Madhi SA, Petersen K, Khoosal M, et al. Reduced effectiveness of *Haemophilus influenzae* type b conjugate vaccine in children with a high prevalence of human immunodeficiency virus type 1 infection. Pediatr Infect Dis J. 2002;21(4):315-21.

120. Gilsdorf JR. Bacterial meningitis in southwestern Alaska. Am J Epid. 1977;106(5):388-91.

121. Singleton R, Bulkow LR, Levine OS, Butler JC, Hennessy TW, Parkinson A. Experience with the prevention of invasive *Haemophilus influenzae* type b disease by vaccination in Alaska: the impact of persistent oropharyngeal carriage. J Pediatr. 2000;137(3):313-20.

122. Losonsky GA, Santosham M, Sehgal VM, Zwahlen A, Moxon ER. *Haemophilus influenzae* disease in the White Mountain Apaches: molecular epidemiology of a high risk population. Pediatri Infect Dis J. 1984;3(6):539-47.

123. Millar EV, O'Brien KL, Watt JP, et al. Epidemiology of invasive *Haemophilus influenzae* type A disease among Navajo and White Mountain Apache children, 1988-2003. Clin Infect Dis. 2005;40(6):823-30.

124. Moulton LH, Chung S, Croll J, Reid R, Weatherholtz RC, Santosham M. Estimation of the indirect effect of *Haemophilus influenzae* type b conjugate vaccine in an American Indian population. Int J Epidemiol. 2000;29(4):753-6.

125. Hammond GW, Rutherford BE, Malazdrewicz R, et al. *Haemophilus influenzae* meningitis in Manitoba and the Keewatin District, NWT: Potential for mass vaccination. Can Med Assoc J. 1988;139(8):743-7.

126. Menzies RI, Singleton RJ. Vaccine preventable diseases and vaccination policy for indigenous populations. Pediatr Clin North Am. 2009;56(6):1263-83.

127. WHO. Global Vaccine Market Report. Available from:

https://www.who.int/immunization/programmes_systems/procurement/mi4a/platform/module2/2019_Global_Vaccine_Market_Report.pdf?ua=1. Accessed August 8, 2020.

128. Chongmelaxme B, Hammanee M, Phooaphirak W, Kotirum S, Hutubessy R, Chaiyakunapruk N. Economic evaluations of *Haemophilus influenzae* type b (Hib) vaccine: a systematic review. J Med Econ. 2017;20(10):1094-106.

129. Muangchana C, Warinsatian P. Incorporation of private demand into cost-benefit analysis of a universal Hib vaccination program in Thailand. Southeast Asian J Trop Med Pub Health. 2011;42(2):376-87.

130. Ozawa S, Clark S, Portnoy A, et al. Estimated economic impact of vaccinations in 73 low- and middle-income countries, 2001-2020. Bull World Health Organ. 2017;95(9):629-38.

131. Malhame M, Baker E, Gandhi G, et al. Shaping markets to benefit global health - A 15-year history and lessons learned from the pentavalent vaccine market. Vaccine X. 2019;2:100033.

132. Peltola H, Kallio MJ, Unkila-Kallio L. Reduced incidence of septic arthritis in children by *Haemophilus influenzae* type-b vaccination. Implications for treatment. J Bone Joint Surg Br. 1998; 80: 471-3.

133. CDC. Nationally notifiable infectious diseases and conditions, United States: Annual tables 2018. Available from: https://wonder.cdc.gov/nndss/static/2018/annual/2018-table2f.html. Accessed June 5, 2020.

134. Bruce MG, Zulz T, DeByle C, et al. *Haemophilus influenzae* serotype a invasive disease, Alaska, USA, 1983-2011. Emerg Infect Dis. 2013;19(6):932-7.

135. Kelly L, Tsang RSW, Morgan A, Jamieson FB, Ulanova M. Invasive disease caused by *Haemophilus influenzae* type a in Northern Ontario First Nations communities. J Med Microbiol. 2011;60(Pt 3):384-90.

136. Tsang RS, Li YA, Mullen A, et al. Laboratory characterization of invasive *Haemophilus influenzae* isolates from Nunavut, Canada, 2000-2012. International journal of circumpolar health. 2016;75:29798.

137. Menzies RI, Markey P, Boyd R, Koehler AP, McIntyre PB. No evidence of increasing *Haemophilus influenzae* non-b infection in Australian Aboriginal children. Intern J Circumpolar Health. 2013;72.

138. Jin Z, Romero-Steiner S, Carlone GM, Robbins JB, Schneerson R. *Haemophilus influenzae* type a infection and its prevention. Infect Immun. 2007;75(6):2650-4.

139. Shoukat A, Van Exan R, Moghadas SM. Cost-effectiveness of a potential vaccine candidate for *Haemophilus influenzae* serotype ‘a’. Vaccine. 2018;36(12):1681-8.

140. Clarke C, Bakaletz LO, Ruiz-Guiñazú J, Borys D, Mrkvan T. Impact of protein D-containing pneumococcal conjugate vaccines on non-typeable *Haemophilus influenzae* acute otitis media and carriage. Expert Rev Vaccines. 2017;16(7):751-64.

141. Santoli JM, Lindley MC, DeSilva MB, et al. Effects of the COVID-19 pandemic on routine pediatric vaccine ordering and administration - United States, 2020. MMWR Morbidity and Mortality Weekly Report. 2020;69(19):591-3.

142. Bramer CA, Kimmins LM, Swanson R, et al. Decline in Child Vaccination Coverage During the COVID-19 pandemic - Michigan Care Improvement Registry, May 2016-May 2020. MMWR Morbidity and Mortality Weekly Report. 2020;69(20):630-1.

143. Close RM, Pearson C, Cohn J. Vaccine-preventable disease and the under-utilization of immunizations in complex humanitarian emergencies. Vaccine. 2016;34(39):4649-55.
